# Designing and evaluating Brain Powered Games for cognitive training and rehabilitation in at-risk African children

**DOI:** 10.1017/gmh.2015.5

**Published:** 2015-05-29

**Authors:** B. Giordani, B. Novak, A. Sikorskii, P. Bangirana, N. Nakasujja, B. M. Winn, M. J. Boivin

**Affiliations:** 1 Department of Psychiatry, University of Michigan, Ann Arbor, Michigan, USA; 2 Departments of Neurology and Psychology and School of Nursing, University of Michigan, Ann Arbor, Michigan, USA; 3 Games for Entertainment and Learning (GEL) Laboratory, Department of Media and Information, Michigan State University, East Lansing, Michigan, USA; 4 Department of Statistics and Probability, Michigan State University, East Lansing, Michigan, USA; 5 Department of Psychiatry, Makerere University, Kampala, Uganda; 6 Departments of Psychiatry and of Neurology & Ophthalmology, Michigan State University, East Lansing, Michigan, USA

**Keywords:** cognition, HIV, malaria, children, rehabilitation, computer games, memory, attention, learning, behavior

## Abstract

**Background.:**

Valid, reliable, accessible, and cost-effective computer-training approaches can be important components in scaling up educational support across resource-poor settings, such as sub-Saharan Africa. The goal of the current study was to develop a computer-based training platform, the Michigan State University Games for Entertainment and Learning laboratory's Brain Powered Games (BPG) package that would be suitable for use with at-risk children within a rural Ugandan context and then complete an initial field trial of that package.

**Methods.:**

After game development was completed with the use of local stimuli and sounds to match the context of the games as closely as possible to the rural Ugandan setting, an initial field study was completed with 33 children (mean age = 8.55 ± 2.29 years, range 6–12 years of age) with HIV in rural Uganda. The Test of Variables of Attention (TOVA), CogState computer battery, and the Non-Verbal Index from the Kaufman Assessment Battery for Children, 2nd edition (KABC-II) were chosen as the outcome measures for pre- and post-intervention testing. The children received approximately 45 min of BPG training several days per week for 2 months (24 sessions).

**Results.:**

Although some improvements in test scores were evident prior to BPG training, following training, children demonstrated clinically significant changes (significant repeated-measures outcomes with moderate to large effect sizes) on specific TOVA and CogState measures reflecting processing speed, attention, visual-motor coordination, maze learning, and problem solving.

**Conclusions.:**

Results provide preliminary support for the acceptability, feasibility, and neurocognitive benefit of BPG and its utility as a model platform for computerized cognitive training in cross-cultural low-resource settings.

Attention and working memory are executive systems of the brain through which a person is able to selectively orient to important information and then store and manipulate that information over a short period. These skills have been suggested to underlie a wide range of complex every-day adaptive abilities, such as reading (Engle *et al*. 1999) and successful decision-making (Hinson *et al*. 2003). In healthy children, for example, working memory and attention issues have been shown to compromise general academic success (Merrell & Tymms, 2001, 2005; Rabiner *et al*. 2004), predict the onset of reading difficulties (Rabiner *et al*. 2000), and detrimentally affect more traditional approaches to academic remediation, such as tutoring (Rabiner *et al*. 2004). Up to 80% of children diagnosed with attention-deficit hyperactivity disorder (ADHD), who demonstrate both attention and working memory deficits have been shown to be at significant risk for poor academic performance (Cantwell & Baker, 1991). They also are at increased risk of not advancing in their grade, being placed in academic support programs, and dropping out of school (Murphy *et al*. 2002; Barkley *et al*. 2006).

When ADHD is diagnosed in children with attention and working memory problems, medication is often the first line of treatment, though long-term and sustainable improvements in academic success have been hard to establish (DuPaul, 2007; MTA Cooperative Group, 2009). Subsequently, parents often discontinue treatment due to cost, side effects, or other issues (Pappadopulos *et al*. 2009). Also, medication treatment for children with attention issues, but without an established diagnosis of ADHD, is not appropriate. Such challenges to these treatment approaches to ADHD are compounded in low-resource settings such as rural Africa.

Some behavioral treatment programs have been developed with reasonably successful positive effects on basic academic performance, but extended outcomes with those approaches also are less clear and maintaining the interventions over time can be very difficult due to high costs and being very labor intensive (DuPaul *et al*. 2002; Barkley, 2007). In response to these concerns and the need for alternative, affordable, and easily sustainable remedial resources, a number of investigators have turned to computer-based training for children with attention and related difficulties, allowing for lower-cost applications for multiple children that can be repeated often with established success. These have been advocated for low-resource settings in Africa (Bangirana *et al*. 2013).

## Brain plasticity

The principle of brain plasticity emphasizes the nervous system's ability to adapt and modify throughout life in order to incorporate new learning and experience into physical and functional change (positive plasticity) as well as disease- or injury-based processes that interfere with performance (negative plasticity) (Mahncke *et al*. 2006). This principle forms the basis for computerized cognitive training. Across the lifespan, the brain, through dynamic changes in structure and function, is able to process and encode new information and use this new learning to effect behavior change, learn new skills, and adapt to new circumstances (Buonomano & Merzenich, 1998; Poldrack, 2000). This principle, based initially on animal research demonstrating macro- and microscopic changes in the brain following training (Buonomano & Merzenich, 1998), has led to the belief that targeted computerized neurocognitive training can successfully be used to make positive changes in ‘real-world behaviors’ for therapeutic interventions (Vinogradov *et al*. 2012).

Neuroimaging studies of young adults have supported this work in positive brain plasticity by showing that computerized cognitive skill training is associated with alterations in gray matter volumes and synaptic activity, which can be quantified using magnetic resonance imaging (Draganski *et al*. 2004; Olesen *et al*. 2004; Pascual-Leone *et al*. 2005; McNab *et al*. 2009; Hoekzema *et al*. 2010). Several of these neuroimaging studies with young adults, for example, provide a neuroanatomical rationale for cognitive training in children with attention and working memory deficits. Markers associated with cognitive training are most evident in prefrontal and parietal areas and in density of cortical dopamine D1 receptors (Olesen *et al*. 2004; McNab *et al*. 2009; Hoekzema *et al*. 2010), areas also associated with ADHD brain abnormalities (Castellanos *et al*. 1996, 2002; Filipek *et al*. 1997).

Cognitive training programs have now been used successfully with children in a range of psychiatric and medical disorders with known cognitive sequelae, including cancer (Hardy *et al*. 2011), cerebral palsy (Akhutina *et al*. 2003), autism (Heimann *et al*. 1995); sensory motor disorders (Sandlund *et al*. 2009), head injury (Hooft *et al*. 2005), and ADHD (Klingberg *et al*. 2002, 2005; Klingberg, 2010; Rabiner *et al*. 2010). Whereas these studies have primarily been carried out in Western populations and higher-economic areas, recent studies have demonstrated that similar successful results with computer-based training can potentially be obtained in improving cognitive issues associated with medical disorders more prevalent in resource-poor settings, specifically cerebral malaria (CM) (Bangirana *et al*. 2009*a*, *b*, 2011) and HIV (Boivin *et al*. 2010) in school-age Ugandan children.

## Challenges to health and cognition in resource-poor settings

Uganda has one of the highest incidences of malaria, worldwide (~480/1000), with about 10% of these cases becoming severe (either severe malaria anemia or CM with coma and often the presence of seizure) (Snow *et al*. 2005; Boivin *et al*. 2007). Over 90% of severe malaria cases worldwide occur in sub-Saharan African children (Snow *et al*. 2005). Cognitive impairment following severe malaria is a well-documented, especially among CM survivors in sub-Sahara Africa (Boivin, 2002; Kihara *et al*. 2006; Kihara *et al*. 2009). Research from Uganda and Senegal has demonstrated a reasonably consistent pattern in these children, consisting of deficits in attention, working memory, and visual–spatial problem solving (Boivin, 2002; Boivin *et al*. 2007; Holding *et al*. 2004; Bangirana *et al*. 2011). There is no successful treatment during acute illness to prevent CM brain-injury effects (Abubakar *et al*. 2007), unfortunately. Over 90% of pediatric HIV infections and AIDS deaths occur in Africa (Foster & Williamson, 2000). Children with HIV have been reported to show subtle and more specific cognitive impairments, including attention problems (Brouwers *et al*. 1994) and visuospatial deficits (Tardieu *et al*. 1995; Fundaro *et al*. 1998; Bisiacchi *et al*. 2000). Executive and working memory deficits have been reported even in neurologically and immunologically asymptomatic children with HIV (Bisiacchi *et al*. 2000). Asymptomatic HIV-infected preschool age children, as compared with healthy children, in Africa have been found to have lower scores on mental processing/attention, sequential processing, spatial memory, and auditory and visual immediate recall (Boivin *et al*. 1995). Anti-retroviral therapy, alone, has not been shown to be effective in reversing these neurodevelopmental consequences (Jeremy *et al*. 2005).

Because the deficit patterns of children with both CM and HIV (e.g. problems in attention, working memory, executive functioning, visual processing) have features in common with children with ADHD and other medical disorders successfully treated with computerized cognitive rehabilitation, our group was not surprised to find similarly good outcomes in early attempts to use computerized cognitive training with children who have survived CM or are have HIV within the Ugandan context (Bangirana *et al*. 2009*a*, *b*, 2011; Boivin *et al*. 2010). Computerized cognitive remediation methods are highly suitable for use in developing or resource-poor settings. In general, computer-based education gaming is scalable and often practical to implement in varied setting and with either a desktop or laptop computer. Support staff or facilitator training requirements are often lower than face-to-face rehabilitative personnel and training tasks are often enjoyable and highly motivating to the participants. With the use of noise cancelling headphones, often multiple individuals can be trained in parallel. Data recording and storage are straightforward and information about game progress is easily attainable and often recorded as both accuracy and speed measures.

## Pilot studies with computerized cognitive remediation – lessons learned

To complete our first studies in Uganda, our group employed a Western-based, popular training system for children. This program set had the advantage of having many different modules covering a number of cognitive domains (e.g. attention, working memory, visual processing, and executive functioning) and difficulty levels, so that it was possible to pick across the modules to determine, based on pre-testing , which appeared to work best in the rural setting. However, there were some disadvantages. Although English is the primary language spoken in Uganda, Luganda, or other regional dialects are spoken most often by children, significantly minimizing the number of options for the training exercises. In addition, many of the topics or stimuli in the programs were far more common to children from Western cultures – such as ‘prizes’ or loud bells and other sounds that would accompany correct performance, pictures and other stimuli from a Western context, as well as the sounds and signs following incorrect answers. Research suggests that such issues potentially reduce the overall beneficial effects of computer-based training (Coffey, 2009). Most game instructions had to be translated by local speakers, as well, to be sure that all children understood the task requirements. In addition, the systems required desktop or laptop computers, generally, leading to issues with easy transport, durability, and cost.

Although new resources are becoming available for reasonable computer purchases for resource-poor settings (e.g. $150, Linux-based laptops under the One Laptop Per Child program), the lack of ability to adapt licensed computerized training programs to tablets or other touch screen devices (increasingly smart phones and tablets) makes using high cost Western-based purchased commercial programs less inviting for large scale-up studies. Battery life and durability of the laptop platform when they often need to be transported in the field and on motorcycles over very rough terrain with inconsistent power options further leads to frustration with reliability of equipment and ability to complete training of multiple children. Finally, a major drawback for most licensed or proprietary programs are their high costs related to development work and other fees, which often make wide scale distribution of such programs in the field too costly for large-scale distribution and leaving these programs for use in the field at schools or other clinical sites. In fact, for many persons and institutions in developing countries, the cost of computer software – from operating systems, to work/office applications, to games – is simply too high to afford, often affecting the ability in these resource-poor settings of finding reasonable solutions to pressing problems (Rajani *et al*. 2003). In addition, Jaeggi *et al*. (2011) have noted that motivation and the level of success in any computer-based training program are tied closely to the generalization of cognitive domain skills beyond simply improving in game performance, and strange or novel programs may not be effective for children from different cultures. In addition, any studies of potential applications for computer-based rehabilitation also need to be considered within a larger context of the emerging use of mobile technologies from sophisticated, high-income settings to across low-income and low-resource areas in the world to address basic health needs. There is a clear need in such cases to move from a proliferation of pilot studies (‘pilotitis’) to demonstrate that interventions can be scaled up effectively and will demonstrate clear improvement in the health system and then to patient outcomes (Labrique *et al*. 2013). Before this can occur effectively in the field; however, there must be careful consideration of the approach that is being ‘borrowed’ or ‘adapted’ from the wider, Western-based computer-training programs.

The Consolidated Standards of Reporting Trials (CONSORT) statement was developed to improve the design and reporting of randomized controlled trials (Eysenbach, 2011) and must be followed once pilot testing is completed and systems are proposed for wide-scale dissemination. However, one of the CONSORT concerns includes the importance of tailoring the intervention to individual circumstances and such resources must include options for users to track their progress and receive feedback in an understandable manner. To meet these challenges in the Ugandan context, our group collaborated with the Michigan State University's (MSU) Games for Entertainment and Learning (GEL) laboratory to develop a prototype of their Brain Powered Games (BPG) program. The goal was to develop a game-based rehabilitation software package that could be used with children with medical disorders with neurological side effects, with an emphasis on children living in areas that are resource poor and lack the needed support for commercially available computerized rehabilitation therapy programs. BPG also was designed within the framework of software globalization (Story, 2004) to represent a computer-based training system that could be adapted, openly to different cultures and languages in collaboration and interaction with local community programmers to aid education and remediation efforts that face roadblocks not only if proprietary software is too costly, but also when software is not produced because a language is not considered ‘commercially viable’ for proprietary software vendors (Rajani *et al*. 2003).

## Development of the BPG platform

The BPG platform was designed as a prototype for effective, culturally sensitive, and well-accepted rehabilitation training software that would be appropriate for field work with children who are from rural settings with the promise of then making these systems available for use in schools and health-related care centers. An important guiding principle in this work was to have a program that could be scaled up to use across multiple platforms, both Apple and Microsoft PCs, as well as tablets, and other touch screen devices, including smart phones and using different underlying operating systems. The ability to scale to a cell phone platform is particularly important given the large projected increase in available networking and cell phone use in Africa (Schumann & Kende, 2013).

**Fig. 1.**
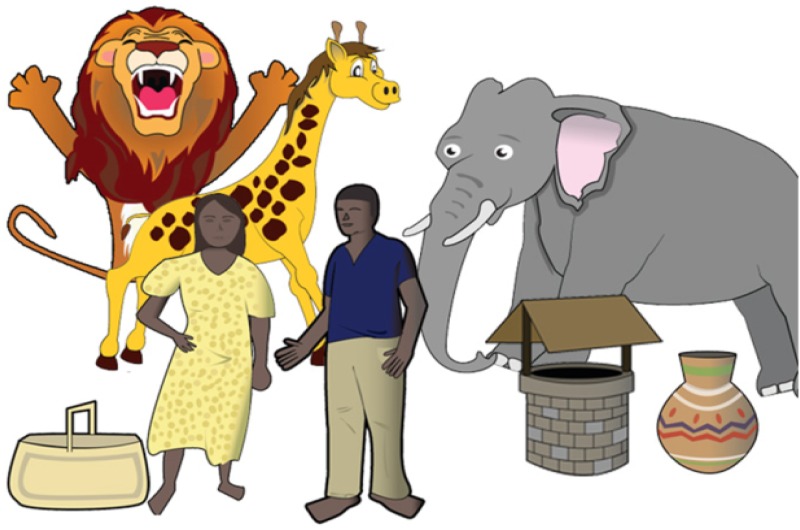
Examples of local Ugandan objects and motif used in the *Themed Visual Design* approach for the development of BPG.

A set of five primary goals led the prototype development for BPG in Uganda. The first, *Themed Visual Design*, helped frame the exercises in the context of children in sub-Saharan Africa by focusing on animals and objects that are native to the area. Inspiration was taken from local African, and specifically Ugandan art and story line that would be recognized and understandable to these younger children. The result was a combination of scenic backdrops with cartoon-like characters that can be incorporated into multiple games (Fig. 1). Principles of *simple mechanics* were incorporated into the design, as children with whom this program would be used generally would have had only limited experience with or access to modern technology. Initial tasks were limited to very simple mouse click or touch screen action that would give children instant feedback and ease the children's sense of complexity. Response demands were slowly increased in complexity as the games went on, starting with a simple tracking game for teaching techniques for responding. More complex game concepts, such as player avatars, were not included based on early pilot testing. More complex response requirements, such as ‘click and drag’ were found to be uncomfortable for many of the children and dropped in favor of simple click responses. The design of the interface also ensures that the demands for understanding game response requirements and game principles are straightforward not only for participants, but also for the trainers.

Currently, research assistants and trainers in-country for our program are undergraduate level psychologists or social workers, though the ease of use of BPG will allow us to evaluate a training protocol for teachers and health care workers, as well as local volunteers. *Custom Data Recording* was designed into the program series to allow for flexible options to allow local sites to decide the most important data to be collected, including flexible input of demographics and other participant information, so this information can be customized for local studies and avoid issues related to local concerns with sensitive data and also to provide the option of avoiding the need for larger central separate data centers. *Server Data Storage* issues also were considered. Many currently available, commercial software training programs store data on the presentation computer, restricting studies to using the same computers or same ‘dongle’ or jump drive for each participant. This limits the ability to back up data, as well as causing issues in the field, where a single computer often can run into reliability issues. Using easily corruptible flash drives or other similar devices to move data can lead to serious difficulty by transferring viruses across computers.

BPG data can be stored on the local presentation device or easily uploaded to an external server when connected to the internet, also reducing the risk of data loss with device failure or theft. Attending to *mobile deployment features* also is important, as this will ensure that programs will (a) run on a wide range of presentation options that use equivalent images and can, in that way, allow the training group to take advantage of new devices with longer battery life; (b) ensure ease of transport with smaller and less heavy devices that can be brought into the field in a bag or backpack on a bicycle or motor scooter; (c) allow for a touchscreen interface with newer screen protectors for simple, natural responding; and (4) be able to take advantage of increasing phone and internet availability throughout Africa.

The aim of the current study was to develop a computer-based training platform, BPG, suitable for use with children within a rural, sub-Saharan Africa setting and then complete an initial field trial with that program. This study would provide support for the acceptability (to children and caregivers), feasibility (in low-resource rural settings), and effectiveness (in terms of neurocognitive benefit) of the proposed intervention by evaluating its use with a group of Ugandan children and comparing their performance on traditional, standardized measures that have already been validated in Uganda, measuring cognitive performance, including focused attention and processing speed, both before and after an extended set of BPG training trials.

## Method

### Participants

The study included 13 boys and 20 girls, diagnosed with HIV/AIDS and residing in a rural town area of Kayunga, Uganda, who were recruited from a group of children who had been passive controls in a previous study of a commercially available computerized cognitive rehabilitation training (CCRT) program (Boivin *et al*. 2010), but had no prior experience with computerized training. Table 1 gives values for descriptors of basic demographics and HIV-related illness. The children had a mean age of 8.6 years [standard deviation (s.d.) 1.8, range 6–12 years of age]. Nineteen of the children (58%) had already been initiated on highly active anti-retroviral therapy (HAART) for at least 6 months and were clinically stable (CD4 > 35%, suppressed viral load, no active opportunistic infections or illnesses) before taking part in this pilot study. All children who began the study completed all the BPG training sessions.

**Table 1.** Descriptive statistics for the BPG sample (N = 33)

Characteristic	Mean (s.d.)
Age (years)	8.55 (2.29)
SES score^a^	30.24 (4.74)
Caldwell HOME score	10.09 (2.95)
HIV RNA (viral load)^b^	7.36 (5.35)
CD4 + Count	37.56 (14.88)
CD4 Activation^c^	6.69 (5.99)
CD8 + Count	49.65 (14.72)
CD8 Activation^d^	20.82 (13.57)
	*N* (%)
Gender	
Male	13 (39)
Female	20 (61)
On HAART at intake	
Yes	19 (58)
No	14 (42)
Recruitment location	
Kayunga County	9 (27)
Kangulumira County	8 (24)
Other	16 (49)
Caregiver	
Mother	10 (30)
Other	23 (70)

HAART, Highly Active Antiretroviral Therapy.

a Socio-Economic Status (SES) Score: Physical Quality of Home Environment Checklist, based on parental literacy and the physical quality of home environment.

b Log 10 viral burden (RNA replication) assessed with HIV-1 Amplicor Monitor Test with plasma separated from EDTA anti-coagulated whole blood.

c CD4 + normalized to CD38 and HLA-DR molecules.

d CD8 + normalized to CD38 and HLA-DR molecules.

### Materials

#### BPG

The BPG package includes five core exercises with plans for expansion in future iterations. Each game includes a visual tutorial, several adjustable settings on the administrative side (e.g. number of trials, increasing speed or accuracy objectives, sounds or stimuli, flexibility in terms of outcome and demographics) and records game play data across a range of options for measuring training-appropriate fidelity (i.e. successful training for each core exercise). Such training success measures have been shown to be important predictors of overall successful transfer of learning to traditional cognitive domains beyond those trained (Jaeggi *et al*. 2011). The design and development of the BPG package was evaluated in an extensive set of preliminary trials that allowed the team to gauge the understandability and appropriateness of the games for Ugandan children, evaluate data recording options and game play, and judge the appropriateness of the stimuli. This time also allowed an evaluation of several other tasks already developed for the original BPG playset (http://brainpoweredgames.msu.edu/science.php) that seemed to be reasonably unbiased in terms of cultural issues and ease of game play.

One of those, Gone Fishing, was incorporated into the final BPG set, based on the fact that all children could complete all aspects of that task training, and its fishing motif was familiar to all participants. It also was clear from this preliminary work that when asked by the research staff, the children enjoyed the games and preferred then to the Western-based options that were also piloted. In addition, in one or two cases, changes had to be made to the game stimuli. For example, children did not mind seeing lions run across the screen in one game, but clearly where distressed in another when a drawing of a lion facing them was one of the features that popped up on the screen (see lion picture in Fig. 1), requiring that stimulus to be removed. Various interfaces were tried with children during this pilot training work. The most optimal approach, initially, appeared to be a very large trackball (approximately the size of a baseball), which was more intuitive than a computer mouse, and this was used for training, as had been used in the previous CCRT study. However as happened in that study, trackballs were found to accumulate dirt over time in their housing and had to be cleaned often or they would become unresponsive, even if optical trackballs were used. Future game iterations will use a touch-sensitive screen, such as a tablet), as this appears to be most optimal interface, and new screen protectors are reasonably priced and easy to clean or remove, allowing ideal response options.

The chosen BPG tasks were as follows. *Butterfly* is a simple game where a butterfly flies across the screen and the player must use the mouse or other input device (including the finger on touch screens and tablets) to click on the butterfly when it stops moving to a random place. The butterfly then starts moving again and the child must click on the next resting point with further repetitions as the child demonstrates familiarity with the type of game interface and response necessary for the training to be successful, reflecting visual tracking, motor control, and simple attention. *iSpy* is a memory game that adapts a common cognitive working memory and learning paradigm, the delayed nonmatch to sample task. The child is asked to look at a scene in which several pertinent items from their environment are pictured (Fig. 1). After a brief display, the picture is removed for a short delay and then re-presented and the child is asked to click on the item(s) that are new that were not in the previous picture of the scene. This task has been shown to relate directly to working memory and learning, by requiring the child to maintain the initial image in mind during the delay and for comparison with the new picture set. As the task progresses more items can be added or some subtracted as the complexity of the demands are increased. In *Stampede*, the child is shown a specific animal to remember. Then, the child sees a group of animals that run across the computer screen and he or she must click only on the animal they had originally been asked to remember. As the rounds progress, the number of animals to remember or the number seen running across the screen increase, again exercising working memory, attention, and visual processing. The *Whacky Animal* training program is adapted from the old style ‘Whack-a-Mole’ games. At the beginning of a round, the child is shown an animal to remember and, as randomly presented animals pop up on the screen, must click or touch only on the correct animal before they disappear back behind the screen. In *Gone Fishing*, the object is to catch as many fish as possible by watching the sequence of bobber movements and then clicking on the bobbers in the same order. The child catches a fish with each correct click, but the sequences get longer with each turn, utilizing short-term memory and attention skills.

### Outcome variables

#### Test of variables of attention (TOVA; Greenberg, 1991)

TOVA is a computer-based measure of sustained attention and vigilance. This test, designed to minimize cultural differences, has been used successfully in Uganda and other African settings in multiple studies (Boivin & Giordani, 2009). It is ideal for such assessment, because it uses large, non-language-based and culture-free simple geometric stimuli (i.e. a large open square with either a solid smaller square at the top or bottom of the open square for each presentation, with only one being selected as the target item and the other the distractor). TOVA is in use in research and clinical settings in over 60 countries around the world to evaluate possible attention problems and discriminate attention disorders from learning disabilities or judge treatment response. The test requires no right–left discrimination and, because of its simple geometric stimuli, has negligible practice effects. A number of scores are provided, including omissions errors (missing the correct target or inattention), commissions errors (identifying the incorrect target as correct or impulsivity), mean response time (speed of processing), and variability in response times (consistency of attention).

#### CogState (Maruff *et al*. 2006)

The computerized cognitive assessment battery, CogState, has been used and validated in clinical research in a variety of cultural settings with children (Mollica *et al*. 2004, 2005). This test uses stimuli consisting of playing cards within a game-like context and is not language dependent, making the assessment engaging and fun, maintaining motivation and reducing potential fatigue effects. The short battery selected for use in this study included: Detection (simple reaction time), Identification (choice reaction time), One-Back card memory (working memory), and One-Card Learning (memory). Card playing is common in Uganda and most children easily recognize the cards and differentiate them with little training. Equivalent stimuli are randomly chosen for each response trial, so repeated assessments can take place with minimum confounding from practice effects (Darby *et al*. 2002; Falleti *et al*. 2006). CogState also includes an option, used in this study, for administering the nonverbal Groton Maze Test (GMLT) to assess basic visuospatial tracking/coordination movement speed (GMLT Chase Test) and planning and executive functioning (GMLT Maze Learning) (Pietrzak *et al*. 2008).

#### Kaufman assessment battery for children – second edition (KABC-2) (Kaufman & Kaufman, 2004)

The KABC-II is a measure of basic cognitive abilities in children aged 3–18 years, designed to minimize the influence of language and cultural knowledge on test results. A special Non-verbal Index, used in this study, allows children to be tested using only gestures to communicate instructions and requires no understanding of English or the need to engage in spoken verbal response. It is the recommended composite cognitive ability measure for the KABC when administered in cross-cultural settings where English is not the preferred language. The earlier version, the K-ABC, has been used across a number of African settings, maintaining its factor structure and demonstrating good construct and predictive validity (Boivin & Giordani, 1993; Boivin *et al*. 1995; Giordani *et al*. 1996; Boivin, 2002; Bagenda *et al*. 2006). The KABC-II has recently been validated with Ugandan children (Bangirana *et al*. 2009*a*, *b*).

### Control variables

Outcome analyses were all adjusted for age, gender, socio-economic status, quality of home environment score, recruitment location, and HAART status (yes/no), based on past research within the sub-Saharan African context indicating the importance of both basic demographic factors, as well as home environment and occupation/income (Boivin & Giordani, 2009). The Physical Quality of Home Environment Checklist (Boivin & Giordani, 1993) is administered to the principal caregiver and scored based on parental literacy and the physical quality of home environment (e.g. water source, type of roofing, living density, cooking facilities, food security, shoes for child, electricity, radio/TV).

### Procedure

Following their involvement as controls in the earlier study, children took part in a 2-month period (24 sessions over 8 weeks) in which they received approximately 45–60 min of training per session, consistent with most recommendations for CCRT training (Bangirana *et al*. 2009; Boivin *et al*. 2010). Once children understood the training instructions for each game, the optimum training times were approximately 5 min for the introductory Butterfly and 10 min for each of the four other tasks. The training programs were developed to be easily administered with minimal language necessary to explain the games and reasonable training options available to be sure that the child understood the game requirements. Once children had experience with the games, basically after one training session, as expected, they could easily sit and begin new training sessions on their own, supporting the possibility of training multiple children at the same time. The BPG platform also were designed to ensure that persons with only minimal basic training in working with children could be trained to set up and run the programs and introduce them initially to children. The training was often completed at one of our clinic sites or in the child's home to avoid issues of stigma that could occur if children were tested within the school setting. All participants were tested after they acknowledged assent and their parents signed informed consents approved by institutional review committees from both Uganda and the USA. Immediately prior to and following the 8-week session, children completed cognitive testing.

### Statistics

Adjusted means of the outcome variables were obtained from linear mixed effects (LME) models with three repeated measures: (1) a time when children were passive controls in the CCRT trial (2 months after intake into the trial), (2) immediately prior to BPG training (5 months after intake into the trial), and (3) immediately post-BPG training. These repeated measures analyses were adjusted for the control variables and the baseline outcome test performance from when a child first entered the original CCRT trial. The explanatory variable of interest was time with three levels: control time, pre-BPG, and post-BPG. The adjusted (least-square) means of each outcome variable were output from the LME model, and the following differences were tested: between the adjusted means of control time *v.* pre-BPG, and the adjusted means pre-BPG *v.* post-BPG. The first comparison of control *v.* pre-BPG captured the effects of time and practice and served as a reference for the second comparison that captured changes that occurred during BPG training. For four of the games, process variables were available, summarizing the average number of correct and missed clicks as well as time to find objects averaged across multiple instances of game playing. These averages were correlated with the outcomes measured post BPG to assess the extent to which the outcomes were related or not related to the success of training.

## Results

Table 2 shows that although there are some variables that demonstrate some improvement over time without intervention (Control *v.* Pre-BPG), changes are clearly evident for select variables following training with BPG (Control *v.* Post-BPG). These BPG-related changes are on measures of attention (TOVA Omissions), processing speed (TOVA Response Time), basic visuomotor tracking speed (GMLT Chase Test), and problem solving (GMLT Learning Test). Effect sizes close to 0.5 are considered moderate, with 0.8 considered large (Cohen, 1988), with several of the change scores demonstrating particularly large effect sizes, consistent with meaningful clinical change. The TOVA percent commission errors (a measure of impulsivity) did not improve significantly as a result of BPG training. Likewise, the KABC-II Nonverbal Index composite score of cognitive ability did not significantly improve as a result of BPG training.

**Table 2.** Adjusted means, s.e.s, and p values and effect sizes for TOVA outcomes at three time points: control, scores at pre- and post-training with BPG

Outcome	Time point	Adjusted mean (s.e.^a^)	*p* value	Effect size
Kaufman-ABC-2 Non-Verbal Index (raw score)	Control *v.*	18.95 (1.10)		
	Pre-BPG	20.92 (1.10)	0.08	0.31
	Post-BPG	21.04 (1.10)	0.91	0.02
TOVA Percent Omission Errors	Control *v.*	24.80 (2.41)		
	Pre-BPG	19.64 (2.41)	0.03	0.37
	Post-BPG	12.35 (2.41)	<0.01	0.53
TOVA Percent Commission Errors	Control *v.*	10.29 (1.52)		
	Pre-BPG	7.78 (1.52)	0.14	0.29
	Post-BPG	10.70 (1.52)	0.09	0.33
TOVA Response Time (ms)	Control *v.*	657.09 (17.22)		
	Pre-BPG	643.76 (17.22)	0.47	0.13
	Post-BPG	565.61 (17.22)	<0.01	0.79
TOVA Response Time Variability (ms)	Control *v.*	259.67 (11.66)		
	Pre-BPG	234.21 (11.66)	0.04	0.38
	Post-BPG	219.27 (11.66)	0.23	0.22
CogState Groton Maze Learning Test Chase Score^b^ (correct moves per second)	Control *v.*	0.23 (0.03)		
	Pre-BPG	0.32 (0.03)	<0.01	0.47
	Post-BPG	0.54 (0.03)	<0.01	1.29
CogState Groton Maze Learning Test Learning Score^c^ (correct moves per second)	Control *v.*	0.09 (0.02)		
	Pre-BPG	0.14 (0.02)	<0.01	0.40
	Post-BPG	0.25 (0.02)	<0.01	1.09
CogState, Detection Time^d^ (log ms)	Control *v.*	2.85 (0.01)		
	Pre-BPG	2.87 (0.01)	0.28	0.25
	Post-BPG	2.85 (0.01)	0.21	0.29
CogState, Identification Time^c^ (log ms)	Control *v.*	2.98 (0.01)		
	Pre-BPG	2.98 (0.01)	0.70	0.07
	Post-BPG	2.98 (0.01)	0.67	0.08
CogState One Card Learning^d^ (accuracy in proportion correct trials)	Control *v.*	0.57 (0.03)		
	Pre-BPG	0.62 (0.03)	0.14	0.33
	Post-BPG	0.67 (0.03)	0.17	0.33
CogState One-Back^d^ (accuracy in proportion correct trials)	Control *v.*	0.65 (0.05)		
	Pre-BPG	0.70 (0.05)	0.17	0.22
	Post-BPG	0.75 (0.05)	0.29	0.17

a Adjusted s.e.s.

b Efficiency of performance: The number of correct moves divided by the time to complete the maze.

c Log of response time in milliseconds.

d Arcsine transformation of correct responses in proportion to all response.

Table 3 further demonstrates the specific nature of these changes, emphasizing that those variables that were significantly improved after BPG training were, for the most part, correlated with BPG process variables for successful training. Variables that did not show significant improvement following BPG training, such as the KABC-II Nonverbal Index composite measure of cognitive ability, did not demonstrate significant correlations with BPG process measures. Although not shown here, all children systematically improved across training trials on the BPG performance measures contained in Table 3 (average number of missed clicks, average number of correct clicks, average time to find target). This indicates fidelity of training in the present preliminary evaluative study of the feasibility of BPG in the field setting with Ugandan children with HIV.

**Table 3.** Correlations of the summary game process variables with baseline-adjusted post-training neurodevelopmental outcomes (outcome improvements from training)

Outcome	BPG Average number of missed clicks *r* (*p* value)	BPG average number of correct clicks *r* (*p* value)	BPG average time to find target *r* (*p* value)
KABC Nonverbal Index (composite nonverbal cognitive ability)	0.20 (0.27)	0.06 (0.75)	−0.08 (0.66)
TOVA Percent Omission Errors	0.41 (0.02)	−0.30 (0.09)	0.32 (0.07)
TOVA Percent Commission Errors	0.19 (0.29)	0.05 (0.78)	−0.13 (0.48)
TOVA Response Time (speed)	0.33 (0.06)	−0.45 (<0.01)	0.40 (0.02)
CogState Maze Chase (speed)	−0.05 (0.76)	0.43 (0.01)	−0.48 (<0.01)
CogState Maze Learning (speed)	−0.19 (0.31)	0.64 (<0.01)	−0.65 (<0.01)
CogState One Card Learning (accuracy)	−0.00 (0.96)	0.26 (0.15)	−0.21 (0.24)

## Discussion

Results of this study demonstrate that it is possible to create a computerized, game-based training platform that is appropriate for children in sub-Saharan Africa regions that are rural and poor in resources and experience with Western-based materials. Children in this study improved their overall speed in processing, attention, and problem solving after BPG training as demonstrated by generalization of learning to performance on standardized test measures. This was in contrast to their previously stable test performance on similar related test measures, but without exposure to computerized training of any type. Similarly, BPG training success measures also were shown to be important predictors of overall successful transfer of learning to traditional cognitive domains beyond simply training on specific computer skills (Jaeggi *et al*. 2011).

The results from this study are significant in demonstrating that CCRT-based programs can be adapted for resource-poor and rural non-Western settings, and that they can be motivated to take part in these training regimens. Our preliminary study also demonstrated the expected improvements in cognitive testing following training, *with neurocognitive performance measures entirely different than the nature of the specific skills needed for BPG performance*. To the casual observer, children seemed to like and be well-motivated to take part in these game-based training exercises. Based on observations during training and when asked afterward, they liked BPG better than the CCRT commercially available software. When asked why, many of the children mentioned that they liked the BPG African-based village setting and sound effects and the use of local animal and village stimuli. This would suggest that improved motivation and learning adherence, which have been associated with increased benefit from computerized training (Jaeggi *et al*. 2011), should provide increased success of BPG in longer, larger scale interventions. In addition, although examiners were present during training and observed that all children maintained training regimens, further research with BPG may demonstrate that it can be easily distributed in the field for children to use on their own at home or in the school setting. Although it is possible that the children also did better with training because of further experience with computers, they did have previous experience with the standardized tests presented on the computer when they were control cases and these results are fairly distinct for specific aspects of executive/attentional control.

The original, initial pilot playtest with a handful of children also showed the importance of prescreening measures to be used with children in cross-cultural research and academic support. This pretest led to a simple, but important change in the game presentation stimuli and also highlighted the fact that cultural differences may certainly exist in terms of understanding and response to stimuli, suggesting the need for careful review of Western-based stimuli in commercial or other Western-based products. Potential issues, for example, include figures related to Halloween (Jaeggi *et al*. 2011) or combat common to many computer games that could provide less than positive experience for some children with resulting potential decline in training benefits. Child interface options also proved important, with the opportunity in the future with having games presented on tablet or smart phone appearing most optimal both in motivating children, as well as in ensuring more straightforward and less problematic response mechanisms. Although there are distinct challenges on adapting available computer training or even other mobile health (mHealth) smart phone or tablet options (Eysenbach, 2011) across cultures, the BPG option does appear successful. Its development can become a prototype or model, particularly as larger CONCUR-based clinical trials studies are undertaken for BPG and other brain-training exercise programs which target-specific cognitive and academic skills.

In the process of developing BPG for the African context, we have learned important lessons that can be helpful when developing computer cognitive games for the American context. When designing local Ugandan objects and motif within the *Themed Visual Design* approach for the development of BPG tasks, we had to be sensitive to differences between the rural and urban or peri-urban environment in the experiences of African children and ensure that items would be equally familiar and acceptable to all participants. The physical and social ecological dynamics can be dramatically different for African children between these two settings. Likewise, we cannot assume a ‘one size fits all’ approach toward the physical and social ecological context of American or European children when designing computer cognitive games. Sometimes it is easier to be sensitized to the importance of cultural context when implementing neurocognitive evaluations and interventions in other cultures, while remaining oblivious to the importance of these factors in one's own culture.

Another important lesson for us pertained to the importance of social relationship and support when implementing a new technology. Our trainers working with the children were Ugandan graduates of bachelor's degree programs in the behavioral sciences at Makerere University in Kampala, Uganda. They were enthusiastic about implementing BPG with an African motif, and that enthusiasm was evident as they presented this training opportunity to the children and their caregivers. This highlighted the importance of having an adult examiner sensitive to when the child was actually ready to begin the training sessions, rather than reliance only on the game parameters of successful learning. Nonverbal cues and concerns of the child in this way could be clearly monitored. The social support engendered by the partnership between African trainer and African child with the consent and favor of African parents was an important dimension to the intervention training program. American practitioners tend to view computer cognitive games in the rehabilitative context as occurring in isolation of the prevailing social context and the support that can be garnered from it. However, as BPG training occurred in the village homes of our study children, the importance of the trainer/parent/child social dynamic was very evident. The social fabric within which the training occurred was likely a vital ingredient to the effectiveness of BPG with these at-risk children who were especially vulnerable psychosocially from the impact of HIV disease on them and their households. It might be easier to overlook the importance of social fabric for more ‘technologically-driven’ interventions in high-resource settings, where the central emphasis is often on the technology itself in isolation from the social fabric within which it is embedded.

Whether in high- or low-resource settings, such skills are vitally important for success in the school, home, community, and workplace for at-risk children, especially impoverished children with HIV as they survive into middle childhood and adolescence because of better access to HAART and other medical care and support. These children need evidence-based interventions accessible and available on a mobile platform at low cost, and readily adaptable to a variety of cultural settings and languages through open access software and collaborative programming. Also, it is important to note that mHealth neurocognitive evaluation and training CCRT systems must be nonproprietary or and readily available for adaptation and implementation within resource-poor areas. BPG is a prototype for just such an intervention. What is needed as a next step is the in-country and local capacity for stakeholders to incubate and evaluate such game packages for scale-up and community-based intervention programs and initiatives. In-country programming and mHealth implementation science teams are best position to foster ongoing improvements for a sustainable program that can meet different and changing situational demands across educational and remediation settings. The ultimate beneficiaries are these generations of children, foundational to the future human capacity and potential for these developing nations in a global economy that is more and more driven by technological advance.
